# An *in silico* drug treatment model to assess the robustness of regional age-based dosing regimens for artemisinin-based combination therapies

**DOI:** 10.1186/1475-2875-11-S1-P91

**Published:** 2012-10-15

**Authors:** Eva Maria Staehli Hodel, Katherine Kay, Daniel Hayes, Anja Terlouw, Ian Hastings

**Affiliations:** 1 Liverpool School of Tropical Medicine, Liverpool, L3 5QA, UK

## 

The standard drug development process for antimalarials and other drugs uses weight-based dosing (mg/kg) to predict blood concentrations of the drug, and hence their effect. Consequently, the current World Health Organization *Guidelines for the treatment of malaria *[1] provide target doses and therapeutic dose ranges in mg/ kg/day. However, in resource-poor settings, age-based dosing is often employed instead of weight-based dosing because of the scarcity of correctly functioning weighing scales outside of clinical settings. Due to the wide variation in weight by age this approach inevitably results in over- and under-dosing of a proportion of the population.

We have recently developed a modelling method to create statistically robust global and regional malaria-specific weight-for-age references representative of the malaria-endemic countries [2] and employed it to predict optimized age-based regimens for artemisinin-based combination therapies (ACTs) for case management of uncomplicated malaria (unpublished). The presented work now assesses the robustness of these age-based regimens using an *in silico* model of antimalarial drug treatment to predict treatment outcome based on individual infection parameters such as parasite numbers, variation in patient pharmacokinetics, and parasite variation in their drug sensitivity [3]. This extended pharmacokinetic/pharmakodynamic model for ACTs allowed us to investigate extreme treatment scenarios in a large number of patients over long follow-up periods that for ethical reasons could not be applied in clinical trials: typical examples include poor adherence (e.g. delayed, reduced or missed doses) or administration of doses above or below recommended therapeutic dose ranges and particularly in most vulnerable individuals such as infants and young children. Pharmacological modelling of antimalarial treatment cannot replace the gold standard of clinical trials, but the model outputs can identify patient groups that are at higher risk of treatment failure due to under-dosing or adverse events due to over-dosing.

We acknowledge the Medical Research Council for funding of this work.
